# Investigation of the RF Sputtering Process and the Properties of Deposited Silicon Oxynitride Layers under Varying Reactive Gas Conditions

**DOI:** 10.3390/ma15186313

**Published:** 2022-09-12

**Authors:** Nikolett Hegedüs, Csaba Balázsi, Tamás Kolonits, Dániel Olasz, György Sáfrán, Miklós Serényi, Katalin Balázsi

**Affiliations:** 1 Institute for Technical Physics and Materials Science, Centre for Energy Research, Konkoly-Thege Miklós str. 29–33, 1121 Budapest, Hungary; 2 Doctoral School of Materials Science and Technologies, Óbuda University, Bécsi str. 96/B, 1030 Budapest, Hungary; 3 Guardian Orosháza Ltd., Csorvási str. 31, 5900 Oroshaza, Hungary; 4 Department of Materials Physics, Eötvös Loránd University, P.O. Box 32, 1518 Budapest, Hungary

**Keywords:** silicon oxynitride, spectroscopic ellipsometry, Berg modelling

## Abstract

In a single process run, an amorphous silicon oxynitride layer was grown, which includes the entire transition from oxide to nitride. The variation of the optical properties and the thickness of the layer was characterized by Spectroscopic Ellipsometry (SE) measurements, while the elemental composition was investigated by Energy Dispersive Spectroscopy (EDS). It was revealed that the refractive index of the layer at 632.8 nm is tunable in the 1.48–1.89 range by varying the oxygen partial pressure in the chamber. From the data of the composition of the layer, the typical physical parameters of the process were determined by applying the Berg model valid for reactive sputtering. In our modelling, a new approach was introduced, where the metallic Si target sputtered with a uniform nitrogen and variable oxygen gas flow was considered as an oxygen gas-sputtered SiN target. The layer growth method used in the present work and the revealed correlations between sputtering parameters, layer composition and refractive index, enable both the achievement of the desired optical properties of silicon oxynitride layers and the production of thin films with gradient refractive index for technology applications.

## 1. Introduction

Pure silicon dioxide (SiO_2_) and silicon nitride (SiN_x_) have long been used as an essential material in electronics technology and are produced by a number of proven technologies [1,2,3]. Their complex form, amorphous silicon oxynitride (SiO_x_N_y_) is a chemically stable coating material with a refractive index of between 1.45 and 2.05, depending on the oxygen and nitrogen content. Due to its advantageous properties, reactively sputtered a-SiO_x_N_y_ could be widely used in optics and optoelectronics. The composition of the deposited layer, and hence its refractive index, is especially sensitive to small changes in sputtering parameters, in particular the oxygen content of the process gas [4]. For this reason, tuning the refractive index of SiO_x_N_y_ would require a sophisticated control of the sputtering process. This is probably the reason why oxynitride is not yet widely used in the technology.

The aim of the present work was to investigate the reactive sputtering process and the physical and technological parameters affecting the properties of SiO_x_N_y_ over the whole composition range from silicon oxide (SiO_x_) to silicon nitride (SiN_x_). Therefore, the full range of composition was deposited under similar experimental conditions by reactive radio frequency (RF) sputtering using the previously introduced combinatorial method [5]. During the experiments, the usual sputtering parameters, such as discharge current and total process gas pressure, were kept constant while oxygen delivery—and thus oxygen partial pressure—were varied by the smart gas inlet technique published previously [6].

The variations of composition and optical properties of the deposited layers along the substrate were characterized by Energy Dispersive Spectrometry (EDS) and Spectroscopic Ellipsometry (SE) measurements. Calculations based on Berg’s model for reactive sputtering enabled the fitting of the deposition temperature and oxygen partial pressure by determining the compositional changes of the SiO_x_N_y_ material obtained experimentally. Thus, the experimental and theoretical analysis of the samples revealed unmeasured and unknown details of the technological process.

## 2. Materials and Methods

### 2.1. Sample Preparation

A series of thin composition-spread, combinatorial amorphous SiO_x_N_y_ layers were prepared by a Leybold Z400 RF sputtering apparatus (Leybold GmbH, Hanau, Germany) at the DC potential range from 1.5 to 1.95 kV. The output of the Advanced Energy CESAR 500 W generator (Advanced Energy Industries, Metzingen, Germany) can be continuously varied between 0 and 500 W on 50 ohms. A matching circuit performed the coupling of the RF power to the target. With this circuit, it was possible to use the total RF power to maintain the plasma without power reflection. A DC voltage was developed on the target (cathode) due to the rectification effect resulted in the difference of the mobility of the ions and electrons. This DC potential was measured directly through a resistance chain capable of isolating the high voltage. The discharge current was calculated from the DC potential and RF power since the inclusion of the matching circuit ensures the coupling of the total RF power into the plasma. The deposition was carried out under gradually varying process gas conditions using a circular silicon target with a diameter of ~76 mm (Kurt J. Lesker, 99.9%) coupled to the RF generator operating at 13.56 MHz. The target and base plate of the equipment were water-cooled. The mechanism including the substrate did not receive separate cooling; therefore, the deposition temperature is considered as the temperature of the processing gas. The substrate was a 25 mm × 10 mm x 0.35 mm germanium (Ge) wafer placed behind a shutter equipped with a 1.5 mm wide slot, which was moved over the substrate by a stepper motor while varying the composition of the process gas. The distance from the target to the substrate was about 50 mm; therefore, the lateral distribution of deposition flux density has negligibly small effect on thickness and composition of the film. All substrates were degreased by acetone with ultrasonic for 3 min, then washed five times in running deionized (DI) water followed by DI water with ultrasonic for 2 min and washed in running DI water. The concentrations of HF (45% HF: DI water = 1:5) was prepared. The substrates were immersed into this HF solution for 1 min, then washed in running DI water, and immediately put into vacuum. The chamber was evacuated to 5 × 10^−4^ Pa pressure prior to the deposition process by means of a turbomolecular pump. Pre-sputtering was carried out for 30 min in a 10:1 mixture of high purity nitrogen (N_2_) and argon (Ar) supplied by conventional gas needle valves resulting in a process gas pressure of 2.6 Pa. The equipment target mounted on a carousel can be rotated relative to the base plate of the sputtering apparatus. During the pre-sputtering, the target was far from the substrate; when the pre-sputtering was started, the target was rotated above the gap covering the substrate. After the pre-sputtering was completed, the deposition of sample was started and, at the same time, a peristaltic pump was switched on to inject oxygen (O_2_) gas from a 48 cm^3^ vial with an initial atmospheric pressure. Peristaltic pumping was maintained for 100 min at a volume flow rate of 1 cm^3^/min that caused a gradual depletion of the vial, resulting in a steady decrease of O_2_ partial pressure in the chamber, between 1 × 10^−2^ and 1 × 10^−3^ Pa. Meanwhile, N_2_ and Ar inflow were kept constant, as described above. The scan time of the slot over the substrate was set to be synchronous with the time of O_2_ injection. As a result, the composition of the deposited layer gradually changed over the whole range from SiO_x_ via SiO_x_N_y_ to SiN_x_.

**Figure 1:**
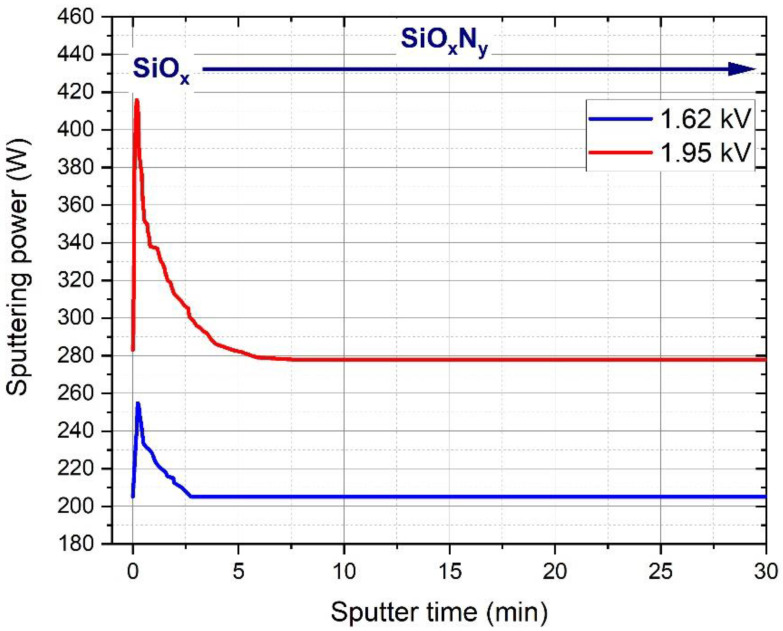
Transient behavior of the sputtering power during the initial stages of the deposition process for the samples sputtered at 1.62 and 1.95 kV voltages.

In order to keep the discharge current constant, the DC potential was kept constant by manually adjusting the RF power. The transient behavior of the sputtering power is plotted in Figure 1, in the cases of samples deposited at voltages 1.62 and 1.95 kV.

At the beginning of the oxygen injection, i.e., at the beginning of the deposition process, the impedance of the plasma decreased; therefore, the sputtering power had to be increased manually to keep the DC potential at a constant level, as can be seen in Figure 1. After the transient phase, the sputtering power became constant over time and a higher power was required to achieve higher DC potential.

### 2.2. Characterization

Energy Dispersive Spectrometry (EDS) measurements were performed by a Scios 2 dual beam Scanning Electron Microscope (SEM, Thermo Scientific, Waltham, MA, USA) equipped with an X-MAX-20 EDS detector (manufacturer: Oxford Instruments, Abingdon, UK) and were evaluated by the software AZtecOne (Oxford Instruments). Electron beam energy was set to 4.2 keV, and the estimated penetration depth was about 200–300 nm (calculations based on pure Si and Ge). Each measurement point corresponds to an area of 200 μm × 137.5 μm, which was investigated for 71 s with a 3.2 nA electron beam.

Spectroscopic Ellipsometry (SE) mapping was carried out along the central axis of the samples using a Woollam M-2000 type ellipsometer (J. A. Woollam Co., Lincoln NE 68508, MA, USA) in the 190–1690 nm wavelength range, at 60, 65, and 70° angles of incidence. The spot size in the ellipsometry measurement was 300 μm, which is small compared to the slot width (1.5 mm) of the shutter and thus the region of varying composition. The acquired data were evaluated by the CompleteEase software (version 5.15., J. A. Woollam Co., Lincoln NE 68508, MA, USA) in the 300–1690 nm wavelength range, using a Brugemann Effective Medium Approximation (EMA) layer on a Ge substrate and an intermix layer between the two as a model. The EMA layer consisted of a Sellmeier type SiO_2_ material and a Cody-Lorentz type SiN_x_ material, whose parameters were fitted at the SiO_x_ and SiN_x_ edges of the sample.

## 3. Results

**Figure 2:**
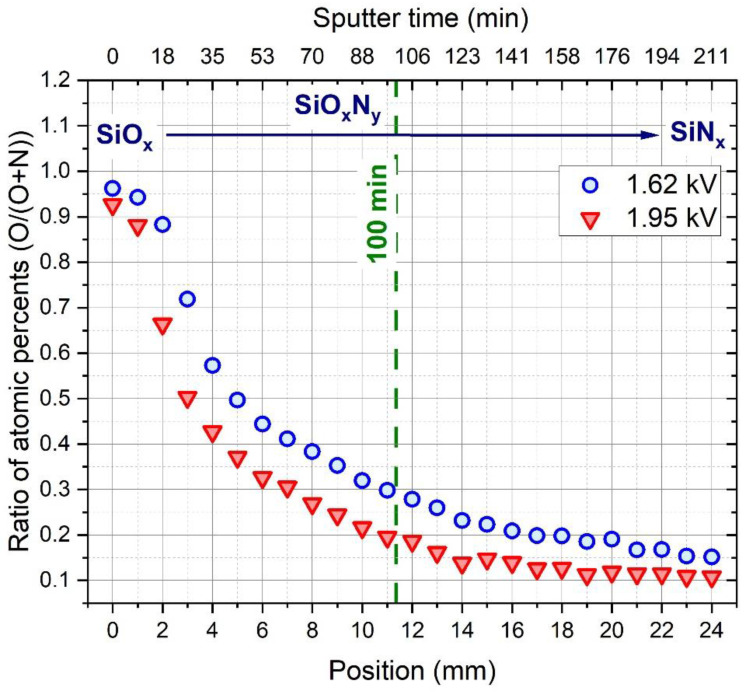
Ratio of atomic concentrations (O/O+N) determined by EDS measurements along the composition spread samples deposited at 1.62 and 1.95 kV DC potential.

**Figure 3:**
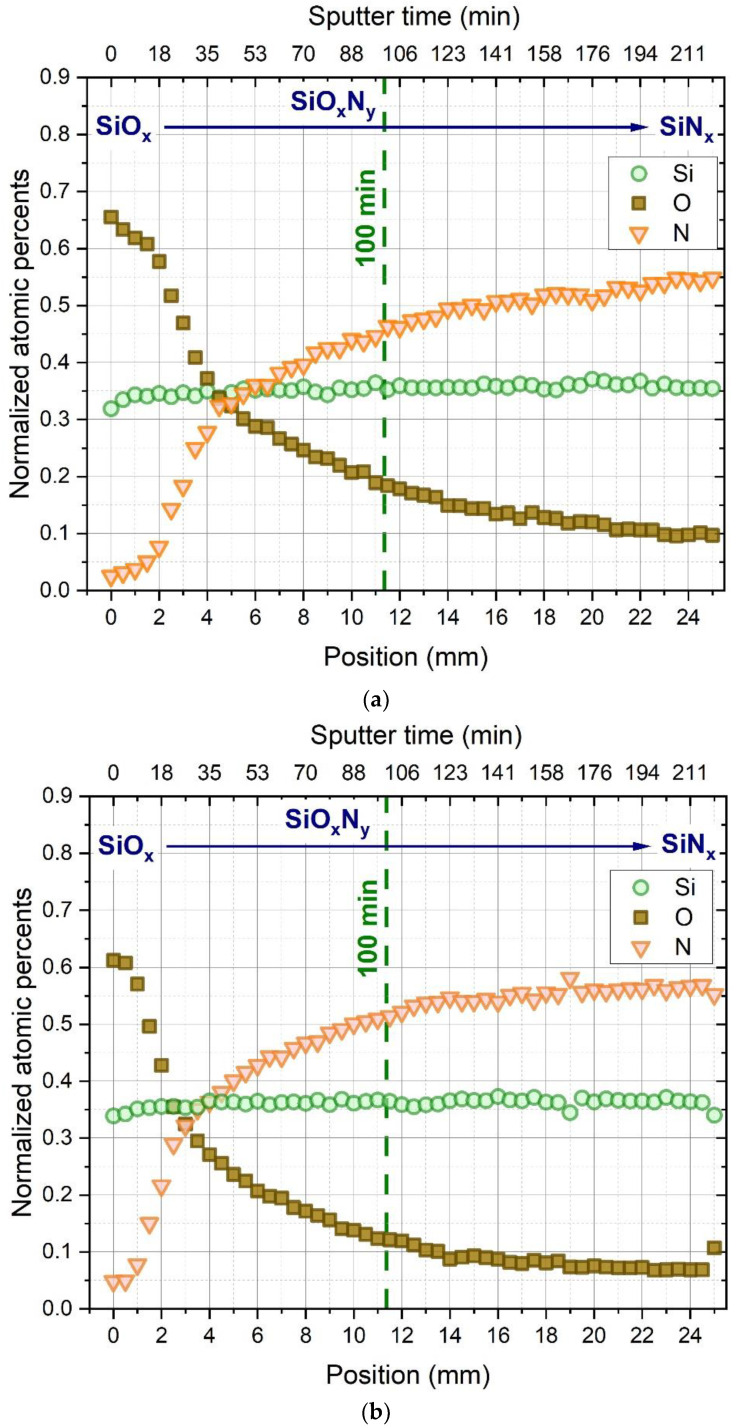
Atomic percentages of Si, N, and O along the composition spread samples deposited at (**a**) 1.62 kV and (**b**) 1.95 kV.

Figure 2 represents the EDS plots of the atomic concentration ratio (O/O+N) of samples sputtered at 1.62 kV and 1.95 kV as a function of distance along the substrate. Both curves show a steady decrease in oxygen and increase in nitrogen content along the samples. Over the entire length of the sample, the layer sputtered at a DC potential of 1.95 kV has a significantly lower oxygen content than that of 1.62 kV, which can be explained by the increased power density. As it is shown on Figure 1, providing 1.95 kV DC potential required the application of higher sputter power and therefore higher power density as a result of the fixed size of the target surface. Then increased power density as known from the literature [7] caused the decrease of the oxygen content of the layer due to the increased reactivity of the nitrogen molecules [8] during the deposition process. Figure 3 shows the relative N, O, and Si content of the samples (excluding the Ge content measured due to the substrate).

In the figures, the dashed line indicates the range in which the Berg model calculations were performed.

**Figure 4:**
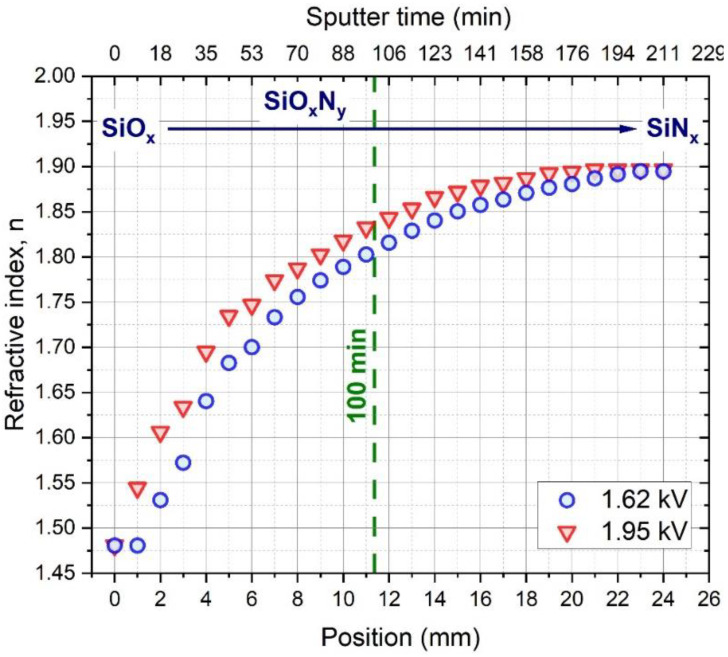
Refractive index (n) of the composition spread samples at 632.8 nm measured along the substrate by spectroscopic ellipsometry.

The refractive index variations of the two samples at 632.8 nm measured by spectroscopic ellipsometry are shown in Figure 4. It is clear that the refractive index increases as the O content decreases, indicating an SiO_x_–SiO_x_N_y_–SiNx transition in the composition spread samples prepared by the experimental process described in Section 2.1.

**Figure 5:**
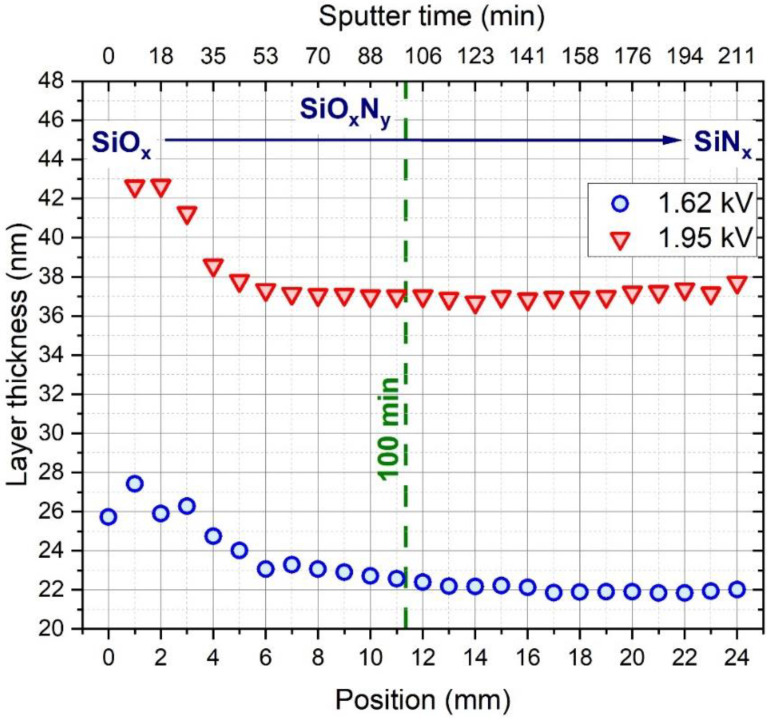
Thickness of the composition spread layers determined by ellipsometry.

The thickness of the deposited layers was also revealed by ellipsometry, as plotted in Figure 5. The layer thickness was highest in the initial high oxygen content layers as a result of the increased sputtering power and thus increased deposition rate presented in Figure 1, and after a rapid decrease, it became roughly uniform. Sputtering at 1.95 kV resulted in ca. 1.7 times thicker layers compared to 1.62 kV, e.g., ~27 and ~42 nm at the SiO_x_ part and ~22 nm and ~38 nm at the SiN_x_ part of the curves, confirming that sputtering rate increases with sputtering voltage.

## 4. Discussion

The Berg model is used for modelling the process of reactive sputtering [9]. The results of the modelling can be used in a variety of ways, from predicting processing behavior to scaling up the deposition and automated control of the manufacturing process [10]. Hegedüs et al. used Berg modelling for the calculations of the hydrogen incorporation into radio frequency sputtered amorphous silicon films [11]. In this work, the Berg model was applied in order to fit the deposition temperature and the partial pressure of oxygen during the reactive sputtering of SiO_x_N_y_ layers. In the Berg model, the target material and the gases involved in the sputter deposition are characterized by three quantities, namely the metal sputtering yield Y_m_, the compound sputtering yield Y_c_, and the sticking coefficient α. The actual values of the material constants in each sample were determined from a fit at the beginning of the variable composition layers, where the partial pressure of oxygen was 8 × 10^−3^ Pa and the deposition temperature was 310 K. The point-by-point changes in both oxygen partial pressure and deposition temperature were fitted using the measured compositional variation of the sample, while knowing the material constants. Although, the Berg model can handle two different reactive gases, we present here a different approach. Our model takes into account a single reactive gas, oxygen, and a composite target, silicon nitride, instead of silicon, given the effect of nitrogen gas applied in the chamber. This approach is based on the two facts that the Berg model considers only the erosion of the top atomic layer of the target and that enough nitrogen gas has been introduced to form silicon nitride over the entire surface of the target. Furthermore, the fact that nitrogen accounts for 90% of the total pressure in the deposition chamber and argon for only 10% also confirms that nitrogen and oxygen can be considered a sputtering and reactive gas, respectively.

The section is structured into two subsections; the equations of the Berg model used for the calculations have been briefly summarized and the fitting results for the material constants, the deposition temperature, and the oxygen partial pressure have been presented, respectively.

### 4.1. Berg’s Model Approach

The reactive gas flow leads to a uniform bombardment of the neutral reactive gas molecules to the inner surface of the deposition chamber. The *F* flux of the molecules in molecules/unit area and unit time can be expressed as follows:

(1) $$F=\frac{p}{\sqrt{2kT\pi m}}$$

where *p*, *k*, *T*, and m refer to the partial pressure of the reactive gas, the Boltzmann constant, the absolute temperature in the processing chamber, and the mass of the reactive gas molecule, respectively.

Since reactions take place between the elemental target atoms and the reactive gas molecules, compound molecules will cover a certain, $\theta_{t}$ fraction of the target surface. This phenomenon is usually called “target poisoning” in the literature [12].

At the same time, the entire target surface (including the $\theta_{t}$ compound fraction and the $\left(1-\theta_{t}\right)$ elemental metal fraction) will be continuously sputter eroded. The rate of target poisoning (compound formation) and the rate of compound sputtering are identical in the equilibrium state, which is expressed by the following equation:

(2) $$\frac{J}{q}Y_{c}\theta_{t}=\alpha 2F(1-\theta_{t})$$

where *J*, *q*, *Y_c_*, and α refer to the ion current density over the target surface, the elementary electronic charge, the sputtering yield of compound molecules, and the probability of the reaction between a reactive gas molecule and a target atom due to collision, respectively.

From Equations (1) and (2), $\theta_{t}$ can be expressed as follows:

(3) $$\theta_{t}=\frac{2\alpha F}{\frac{J}{q}Y_{c}+2\alpha F}$$

(4) $$\theta_{t}=\frac{2\alpha \cdot \frac{p}{\sqrt{2kT\pi m}}}{\frac{J}{q}Y_{c}+2\alpha \cdot \frac{p}{\sqrt{2kT\pi m}}}$$

As a result of the above, the sputter-eroded material from the target surface can be devided into two groups: (i) compound material sputtered from the $\theta_{t}$ fraction, and non-reacted target atoms sputtered from the $\left(1-\theta_{t}\right)$ fraction of the target. *F_c_* and *F_m_* refer to the total number of eroded molecules or atoms from the $\theta_{t}$ and the $\left(1-\theta_{t}\right)$ fraction of the target, respectively, and can be expressed by the following equations:

(5) $$F_{c}=\frac{J}{q}Y_{c}\theta_{t}A_{t}$$

(6) $$F_{m}=\frac{J}{q}Y_{m}(1-\theta_{t})\;A_{t}$$

where $A_{t}$ and $Y_{m}$ refer to the total area of the target surface and the metallic sputtering yield, respectively.

In the Berg model’s approach, the term “collecting surface” is used to refer to the *A_c_* total surface area (including the substrate) that receives sputtered material during the deposition. All eroded materials from the target are assumed to be uniformly deposited on the entire collecting surface.

**Figure 6:**
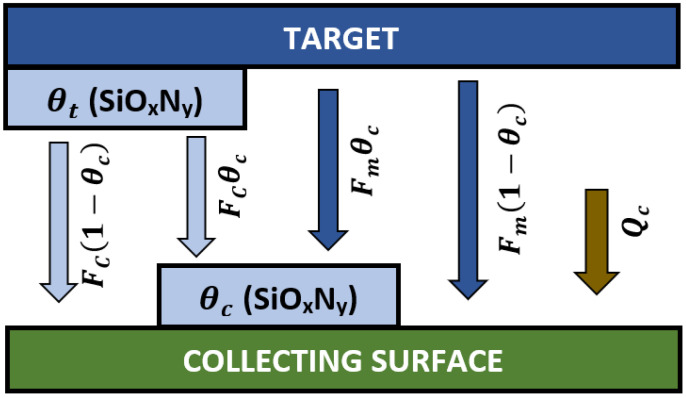
Schematic view of the material fluxes resulting SiO_x_N_y_ formation on the collecting surface.

The material deposition on the collecting surface is considered to be the result of five different material fluxes, as is presented in Figure 6.

The *F_c_* compound material flux eroded from the $\theta_{t}$ part of the target leads to compound formation on the $\theta_{c}$ compound part and $1-\theta_{c}$ metallic part of the collecting surface. Similarly, the *F_m_* flux of eroded metallic material promotes metallic deposition on the $\theta_{c}$ and $1-\theta_{c}$ parts of the collecting surface.

In addition to the target erosion, compound formation by reactions between the reactive gas molecules, and the non-reacted atoms on the collecting surface will also contribute to the layer growth on the collecting surface. These reactions will consume *Q_c_* amount of reactive gas molecules, which can be expressed by as follows based on Figure 6:

(7) $$Q_{c}=\alpha F(1-\theta_{c})A_{c}$$

where $\theta_{c}$ refers to the compound fraction of the collecting surface.

From the material fluxes presented in Figure 6, *F_c_*$\theta_{c}$ does not change the stoichiometry of the layer, and thus the value of $\theta_{c}$, since this material flux refers to compound material erosion from the target to the $\theta_{c}$ part of the collecting surface which is already in a compound state. Similarly, *F_m_*$\left(1-\theta_{c}\right)$ will also not change the value of $\theta_{c}$ due to the metallic material erosion to the $\left(1-\theta_{c}\right)$ metallic part of the collecting surface. On the other hand, *F_m_*$\theta_{c}$ will decrease, while *F_c_*$\left(1-\theta_{c}\right)$ and Q_c_ will increase the value of $\theta_{c}.$

Assuming steady state at the collecting surface, contributions to the increase and decrease of $\theta_{c}$ should be identical, which can be mathematically expressed by:

(8) $$2Q_{c}+F_{c}(1-\theta_{c})=\theta_{c}F_{m}$$

In Equation (8), *Q_c_* is multiplied by two based on the fact that one reactive gas molecule contains two reactive gas atoms; therefore, one reactive gas molecule will contribute with two compound molecules to the increase of $\theta_{c}.$

From Equations (1) and (2) and Equations (5)–(8), $\theta_{c}$ can be solved as a function of the *p* partial pressure of the reactive gas and the *T* deposition temperature:

(9) $$\theta_{c}\left(p,T\right)=\frac{2\alpha FA_{c}+\frac{J}{q}Y_{c}\theta_{t}A_{c}}{\frac{J}{q}A_{t}\left(Y_{m}+\theta_{t}\cdot \left(Y_{c}-Y_{m}\right)\right)}$$

From Equations (3) and (9) $\theta_{c}\left(p,T\right)$ can be expressed in the following form:

(10) $$\theta_{c}\left(p,T\right)=\frac{2\alpha FA_{c}+\frac{J}{q}Y_{c}A_{c}\cdot \frac{2\alpha F}{\frac{J}{q}Y_{c}+2\alpha F}}{\frac{J}{q}A_{t}\left(Y_{m}+\frac{2\alpha F\cdot \left(Y_{c}-Y_{m}\right)}{\frac{J}{q}Y_{c}+2\alpha F}\right)}$$

The *J* ion current density over the target was calculated based on the following equation [13]:

(11) $$J=\frac{P}{U\cdot A_{t}}$$

where *P* and *U* refer to the measured power and effective voltage, respectively.

In the following subsections, we present the results of two fitting processes, aiming to fit (i) the *Y_m_*, *Y_c_*, and α material constants and (ii) the O_2_ partial pressure and the *T* temperature based on the Berg model approach. In the case of all constants fitting, the $\theta_{c}^{exp}$ experimental value of $\theta_{c}$ and the $\theta_{c}^{Berg}$ modelled value of $\theta_{c}$ were compared at certain points of the sample. $\theta_{c}^{exp}$ was determined from EDS measurements based on the following equation:

(12) $$\theta_{c}^{exp}=\frac{\left[O\right]}{\left[O\right]+\left[N\right]}$$

where [*O*] and [*N*] refer to the concentration of oxygen and nitrogen, respectively.

$\theta_{c}^{Berg}$ was calculated from Equation (10), where *A_t_* and *A_c_* were considered to be 44 cm^2^ and 80 cm^2^, respectively.

During the fit, the mean squared error between $\theta_{c}^{exp}$ and $\theta_{c}^{Berg}$ was minimized by varying the free parameters of $\theta_{c}^{Berg}$. Since two different fitting processes were performed, we describe the used measurement points and the free parameters for both fittings separately in Section 4.2 and Section 4.3.

### 4.2. Fitting of the Material Constants

At the beginning of the four SiO_x_N_y_ combinatorial layer deposition, the values of *T* (310 K) and p (8 × 10^−3^ Pa) are known. Therefore, the first measured points of the four samples were used to fit the *Y_m_*, *Y_c_*, and α material constants. The value of F was calculated from Equation (1), knowing T and p. The J ion current density was calculated based on Equation (11) from the measured values of the RF power and the DC potential presented in Section 2.1. Knowing *p*, *T*, *F*, *J*, *A_t_*, and *A_c_*, the only unknown parameters in Equation (10) is *Y_m_*, *Y_c_*, and α, which were considered to be free parameters. During the fit, these free parameters were varied in order to minimize the mean squared error between the four $\theta_{c}^{exp}$ data and the corresponding $\theta_{c}^{Berg}$ values. 

**Figure 7:**
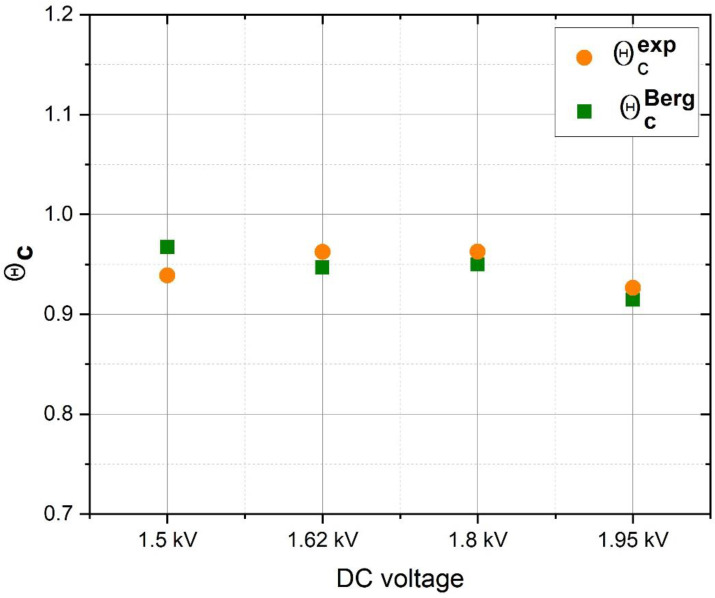
Experimental and Berg modelled data of the first point of the samples at various DC potentials.

Figure 7 presents $\theta_{c}^{exp}$ data and the corresponding $\theta_{c}^{Berg}$ values obtained from the fit results.

The fitting results of the *Y_m_*, *Y_c_*, and α material constants are reported in Table 1.

The fitted value of *Y_m_* (0.7) is very close to the value reported in the literature [14] for nitrogen (~0.5–0.6). The resulted value of α is also in the range of its typical reported value (0.5–1) [10]. However, we are not aware of any reported data in the literature for *Y_c_* for nitrogen in case of SiO_x_N_y_ compound material, and the fact that the resulting *Y_c_* value is lower than the *Y_m_* is in good alignment with the known correlation that *Y_m_* is always higher than *Y_c_* [15].

The good alignment between the $\theta_{c}^{exp}$ and $\theta_{c}^{Berg}$ values as well as the agreement between the obtained material constants and their values reported in the literature prove that the Berg model equations of Section 4.1 are suitable for describing the reactive sputtering process of oxygen-rich SiO_x_N_y_ layers.

### 4.3. Fitting of Temperature and Oxygen Partial Pressure

The variations of p and T during the deposition of SiO_x_N_y_ layers sputtered with 1.62 and 1.95 kV were determined by the second fitting process. However it is known that the values of *Y_m_, Y_c_*, and α could vary slightly under different gas processing conditions; in the application of the Berg model approach they are usually considered to be constant [10], characterizing the affected materials and sputtering equipment independently from the actual process parameters. Based on this assumption, the values of *Y_m_, Y_c_*, and α were considered to be the values reported in Table 1 throughout the entire deposition process of all samples. Similar to the first fitting process, the value of J at each measurement point of the samples was calculated based on Equation (11) from the measured RF power and the DC potential presented in Section 2.1. Knowing *Y_m_, Y_c_*, and α and *A_t_* and *A_c_*, as well as *J* at a certain point of the sample, $\theta_{c}^{Berg}$, referring to the stoichiometry of the layer at the affected point, can be calculated as a function of *p* and *T* based on Equations (1) and (10). Varying *p* and *T* as free parameters, the difference between $\theta_{c}^{Berg}$ and $\theta_{c}^{exp}$ at the affected point can be minimized. Based on this method, *p* and *T* were fitted individually in each measured points of the samples sputtered with 1.62 and 1.95 kV. 

**Figure 8:**
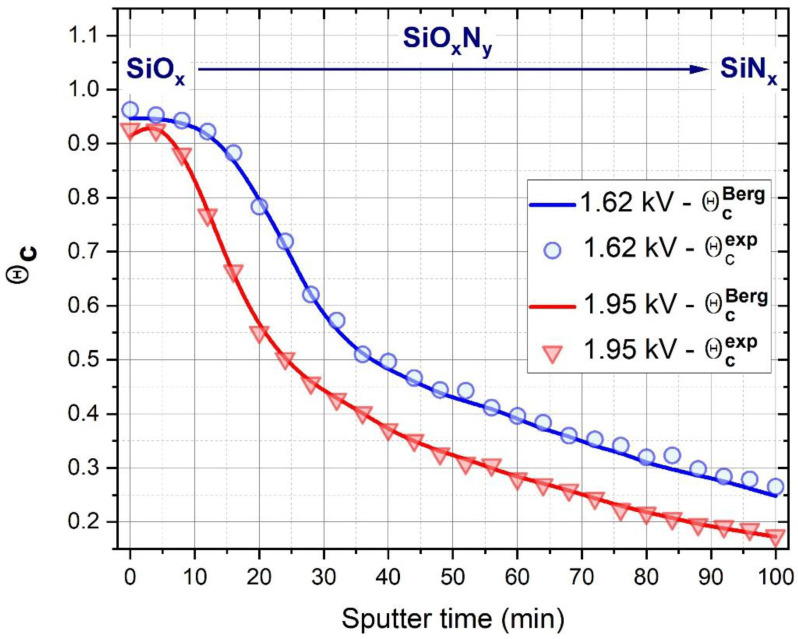
Experimental and Berg-modelled data of the compositional change of the deposited layers as a function of sputter time.

Figure 8 represents a very good agreement between the fitted $\theta_{c}^{Berg}$ and $\theta_{c}^{exp}$ data in case of all points of the samples, proving that the introduced Berg model equations are able to correctly follow the lateral compositional variation of the samples.

**Figure 9:**
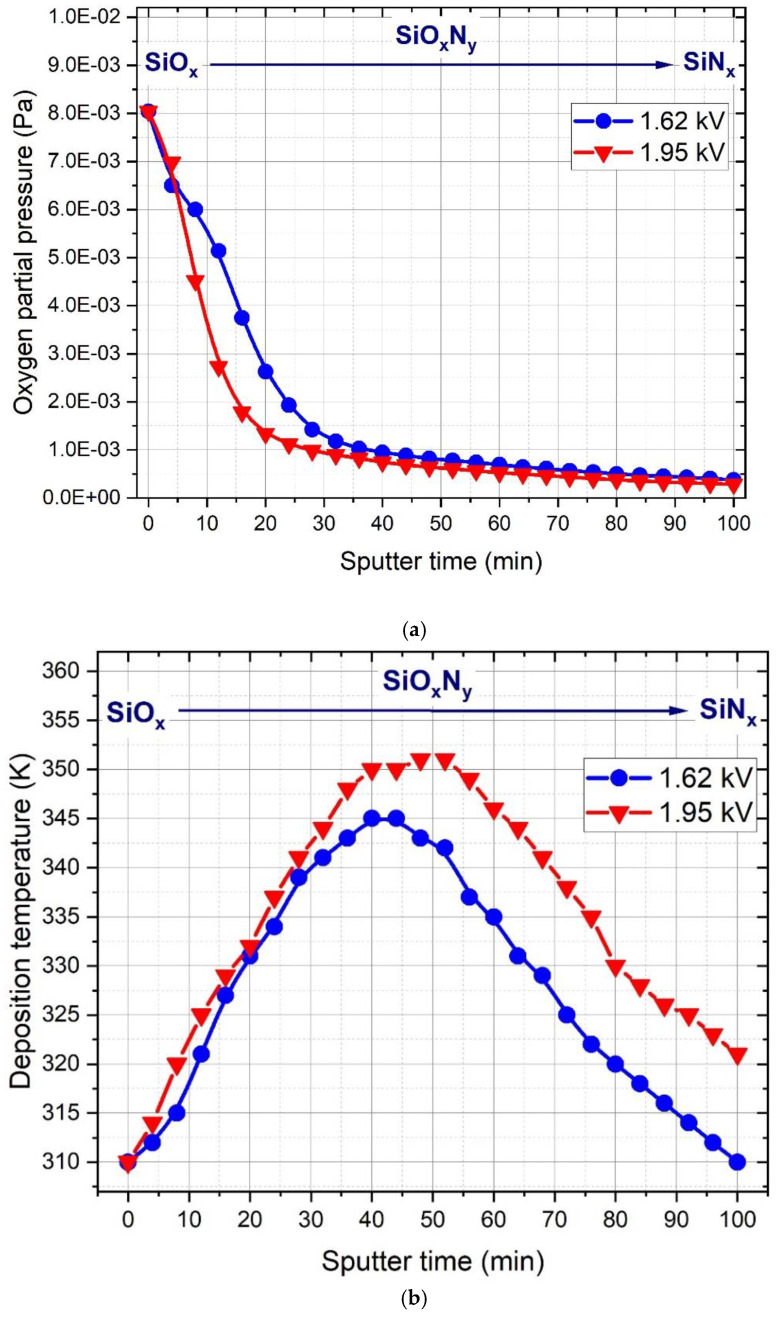
Fitting results of the (**a**) oxygen partial pressure and (**b**) deposition temperature defined in Equation (1) as a function of sputter time.

Figure 9 shows the variation of the fitted oxygen partial pressure and the deposition temperature as a function of sputter time. Essentially, the shape of the oxygen partial pressure functions (Figure 9a) follows the characteristic behavior of the peristaltic pump [6] applied to the oxygen injection during the deposition. For both samples, the variation of the fitted O_2_ partial pressure is in a good agreement with the lateral compositional variation measured by EDS (Figure 2).

Figure 9b represents the fitted T values of the deposition process carried out at 1.62 and 1.95 kV DC potential. At the beginning of O_2_ injection, T increases regardless of the applied DC potential and reaches its maximum after about 45 min of sputtering. Such an increase in temperature could be attributed to the exotherm heat of oxidation [16,17]. It is also supported by the fact that after ~45 min of sputtering, *T* starts to decrease in agreement with the reduced O_2_ partial pressure shown by Figure 9a. The maximum of *T* for the process carried out with 1.62 kV was ~345 K, while increased DC potential, and thus increased RF power, resulted in a higher maximum of the temperature (~350 K), which suggests that the effect of the oxidation on the temperature defined in Equation (1) was more pronounced when a higher DC potential was applied.

## 5. Conclusions

All things considered, from the deposition parameters and the composition of the grown layers, we were able to infer parameters we did not measure, such as partial pressure and temperature. The Berg model was used to interpret the experimental results. The material constants of the Berg model were deduced from the fitting results of the first measurement points of the different combinatorial layers.

A new approach to the Berg modelling of the SiO_x_N_y_ sputtering process was adopted, where the metallic Si target sputtered with a uniform nitrogen and variable oxygen gas flow was considered as an oxygen gas-sputtered SiN target. This application of the Berg model could be very useful in the future for modelling any reactive sputtering process where the amount of reactive gas injected into the chamber leads to the formation of a thicker layer of compounds on the surface of the target, instead of the very thin layer assumed by the original approach of the Berg model. To our knowledge, this is the first time that such an approach to Berg modelling has been reported in the literature.

The layer growth method used in the present work and the revealed correlations between sputtering parameters, layer composition, and refractive index enable both the achievement of the desired optical properties of silicon oxynitride layers and the production of thin films with gradient refractive index for technology applications.

## Figures and Tables

**Table 1:** Fitting results of the *Y_m_*, *Y_c_*, and α material constants.

Parameter	Result
*Y_m_*	0.7
*Y_c_*	0.3
*α*	0.6

## Data Availability

Not applicable.
